# Conceptualization of gender in published malaria and gender research: a systematic descriptive review

**DOI:** 10.1186/s12939-025-02545-9

**Published:** 2025-07-23

**Authors:** Deborah Atobrah, Benjamin K. Kwansa, Patience G. Okyere-Asante, Abena Kyere, Delali M. Badasu, Irene A. Kretchy

**Affiliations:** 1 Centre for Gender Studies and Advocacy, University of Ghana, Legon, Ghana; 2 Institute of African Studies, University of Ghana, Legon, Ghana; 3 Regional Institute of Population Studies, University of Ghana, Legon, Ghana; 4 School of Pharmacy, University of Ghana, Legon, Ghana

**Keywords:** Gender conceptualisation, Malaria research, Descriptive review, Sex-disaggregated data, Gender analysis, Malaria and gender

## Abstract

**Background:**

Malaria disproportionately affects vulnerable and marginalised population subgroups, including women and girls, migrants, and persons with disabilities. Gender roles expose men and women differently to malaria risks. Similarly, restrictive gender norms pose unique challenges to women and girls in accessing preventive treatment and care. Gender norms that perpetuate hegemonic masculinity also expose men and boys to malaria, resulting from occupational exposure and untimely access to malaria treatment and care. Unfortunately, the gender dimensions of malaria remain under-researched. This systematic descriptive review examines how gender has been conceptualised in published malaria and gender research over the last three decades.

**Methods:**

The keywords “malaria AND gender” were used to search for articles published in English from 1995 to 2024 in four databases (PubMed, Scopus, Science Direct, and Google Scholar). The Preferred Reporting Items for Systematic Reviews and Meta-Analysis (PRISMA) was adopted for this review. The Rayyan intelligent systematic review software was used to collate, manage, and screen articles retrieved from the search engines. The gender analysis matrix advanced by Morgan and colleagues was used to analyse the conceptualisation of gender in published malaria and gender research.

**Results:**

A total of 57 published articles that met the inclusion criteria were included in the final review. We found that the majority of the published papers on malaria and gender have been biomedical in nature, consequently reducing gender analysis to only sex-disaggregated data. Moreover, most of the studies employed a quantitative research approach, with the majority being laboratory-based research, focussing on sub-Saharan Africa.

**Conclusion:**

There is a need for more social science research that employs qualitative, mixed-methods, and community-based approaches to malaria and gender research. These approaches extend gender analysis beyond sex and/or gender-disaggregated data, and includes other domains, such as access to resources; distribution of labour; practices and roles; norms, values and beliefs; and decision-making power.

## Background

Malaria remains a global health challenge, especially in low- and middle-income countries (LMICs). According to the 2024 World Malaria Report, there were 263 million cases of malaria in 2023 compared to 252 million cases in 2022 [1]. The estimated number of malaria deaths marginally declined from 600,000 in 2022 to 597,000 in 2023 [1]. Children under five years accounted for 80% of malaria deaths in the WHO African region [1]. Although malaria has important gender dimensions because pregnant women [2, 3], children under five years [4], and adolescent girls [5, 6] suffer disproportionately from the malaria burden in the African region, malaria research has paid little attention to malaria and gender intersection. Men and women have differential biological and sociocultural risks to and impacts from malaria. For instance, adolescent girls and women are more susceptible to malaria infections during pregnancy [3, 6, 7]. They also carry the burden of caring for household members with malaria [1]. Restrictive gender norms pose unique challenges to women and girls in accessing preventive treatment and care [8]. Gender norms, which perpetuate hegemonic masculinity, on the other hand, expose men and boys to malaria, resulting from occupational exposure and delayed access to malaria treatment and care [1, 9, 10]. However, the gender dynamics of malaria, that is, how malaria affects women and men, and girls and boys, differently because of their socially assigned roles, responsibilities, and rights, are under-researched as gender remains a critical missing lens in malaria research. Unsurprisingly, it was the most recent World Malaria Report (WMR) that laid an overt emphasis on this relationship between Malaria and Gender [1], highlighting how women and men, as well as girls and boys, are differentially vulnerable to malaria. However, understanding the gender dimensions of malaria and applying a gender lens to health research and programming can improve health outcomes while also enhancing gender equality [11].

Malaria and gender research have not sufficiently “integrated sex and gender analysis of the differences in exposure risks, disease susceptibilities, prevention and treatment behaviours, and preferences in product design” [12]. However, research that integrates a gender lens reveals the differential risks, presentation, effects, health-seeking behaviours, and social implications of diseases on men and women [13]. Most of the materials on malaria and gender have been largely policy documents and grey literature [12, 14–16]. Evidence from published academic research, however, remains crucial in contributing to the knowledge required for policy formulation and strategic investments in malaria control and elimination.

There is also a tendency for researchers to use sex and gender interchangeably in malaria and gender research. While sex generally refers to the biological differences between males and females based on hormones, sex chromosomes, internal reproductive organs, and external genitalia, gender refers to the social construction of the roles, culturally prescribed responsibilities and rights, attitudes, and behaviours of males and females [14, 17]. Sex-disaggregated data remains an important starting point for gender analysis in malaria and gender research. Gender analysis, in addition, encompasses domains such as access to resources, distribution of labour, practices and roles, norms, values and beliefs, decision-making power and autonomy, policies, laws, and institutions [18].

Moreover, while the biomedical dimensions of malaria have received substantial attention, there is growing recognition that social determinants, including gender, play a critical role in shaping malaria vulnerability, exposure, prevention, and treatment outcomes [19]. However, the conceptualization and operationalization of gender within malaria research remain uneven and, in many cases, underdeveloped or unexamined [20]. Additionally, little is known about the geographic distribution and methodological approaches used in malaria and gender research. Meanwhile, data on such geographic distribution provides insight into the contexts in which gendered analyses of malaria occur, potentially revealing under-researched regions where gendered dynamics of malaria are nonetheless critical. Similarly, an understanding of methodological trends highlights prevailing approaches and reveals opportunities for innovation and more inclusive research frameworks [21].

This review examines the existing body of published research on malaria and gender to investigate how gender has been conceptualised and applied in these studies over the past three decades. It aims to show clearly how the research community has used the various domains of gender analysis in malaria and gender research. The review seeks to answer the following questions:

1. How has gender been conceptualized and applied in published malaria and gender research?
2. How have published malaria and gender research applied key gender concepts/gender analysis domains such as sex-disaggregated data, access to resources, distribution of labour, practices and roles, norms, values and beliefs, decision-making power and autonomy and policies, laws and institutions?
3. What disciplines and geographic regions have malaria and gender research been most published?
4. What have been the dominant methodological approaches in published malaria and gender research?

## Methodology

This study is a systematic descriptive review that describes how gender has been conceptualized or applied in published malaria and gender research. The purpose of descriptive reviews is to “determine the extent to which a body of knowledge in a particular research topic reveals any interpretable pattern or trend with respect to pre-existing propositions, theories, methodologies or findings” [22, 23]. The study examined how gender has been used in selected published studies on malaria and gender, specifically in terms of definitions, gender concepts, and terminologies applied. In line with the guidelines for descriptive reviews, structured methods were employed in selecting studies for the review and analysis [22, 23]. We paid attention to the fields or disciplines of study, the research methods used, the year of publication, the study findings, and the conclusions. We focused on published research only, excluding grey literature and unpublished research. This study, therefore, does not include theses, conference proceedings, policy documents, and reports. The review focused on available published research on malaria and gender over the last three decades (1995–2024).

### Inclusion criteria

- All existing published research (peer-reviewed articles) on malaria and gender over the last three decades (1995–2024) were included in the review.
- Only published social science papers with both malaria and gender in their titles were included in the review.
- Only articles in English were included.
- There was no geographical limitation in terms of the spread of publications.

### Exclusion criteria

- Articles that were not written in English were excluded.
- Articles with only malaria or gender in the title were not included.
- Unpublished manuscripts, policy documents and reports, thesis, conference proceedings, datasets, and presentations were excluded from the review.

### Search strategy

The keywords “gender AND malaria” were exclusively used for article searches from Scopus, Science Direct, and Google Scholar. The search was limited to article titles only. The focus of the study was to examine how authors who sought to do a gender analysis of malaria conceptualised gender, hence the use of “gender” and not “women” or “men”. It is acknowledged that this approach may result in missing other studies that may have used women, men, or both, while applying a gender lens. A total of 156 records were identified: 99 from Google Scholar, 27 from Scopus, 23 from PubMed, and seven [7] from Science Direct. All these records were imported into the Rayyan Intelligent Systematic Review Software and screened for duplication. Seventy [70] duplications were detected and excluded, and full-text screening was conducted for the remaining 86 records, one of which could not be retrieved. Twenty-eight [28] records were excluded based on the inclusion and exclusion criteria: policy documents and reports [9], thesis [5], conference proceedings [5], unpublished manuscripts [4], datasets [2], presentation [1], non-English articles [2] were thus excluded. A total of 57 published articles were included in the final analysis. The review is registered in the open science framework (ref ID: https://osf.io/ve7ur/).

**Fig. 1:**
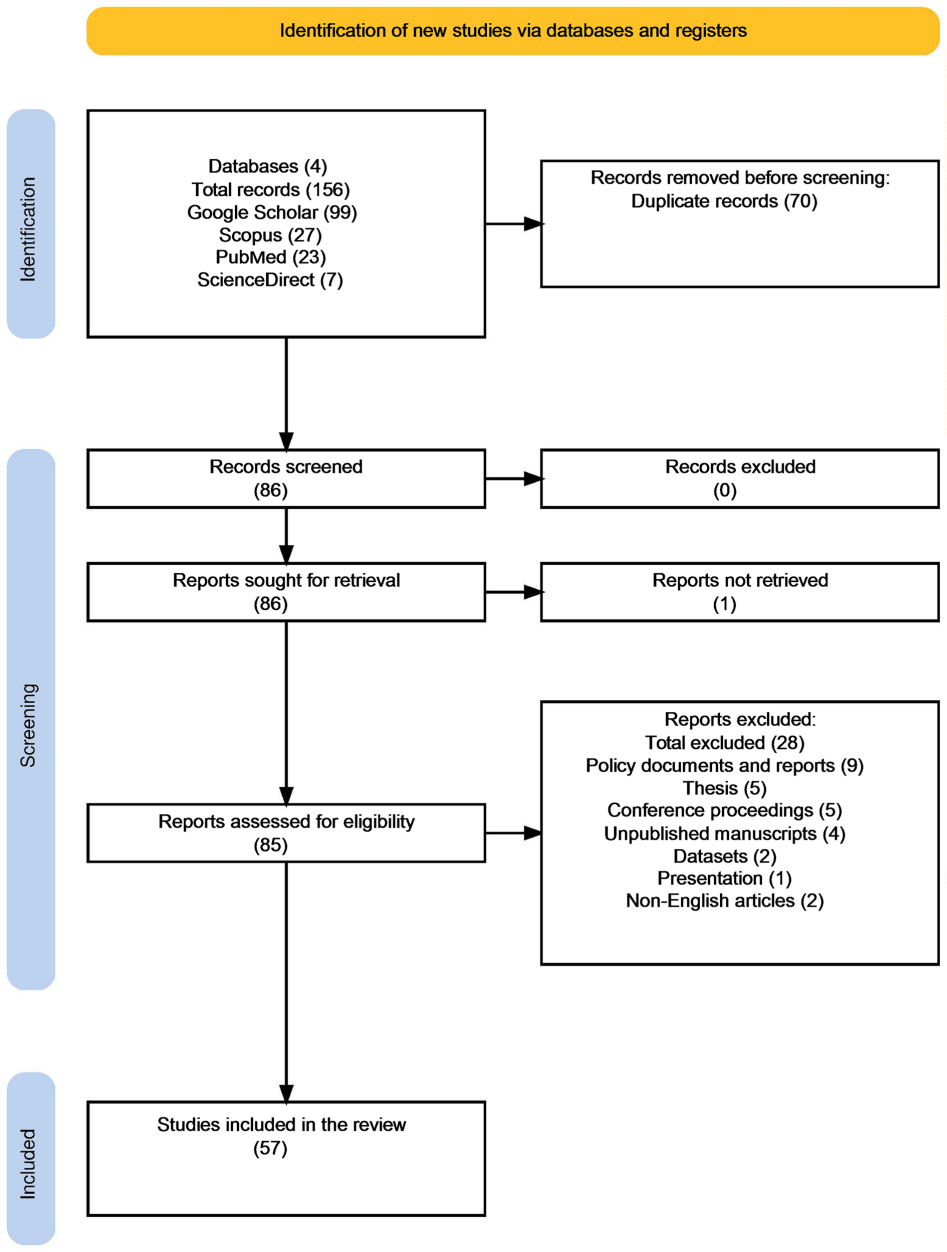
PRISMA flow chart for the identification, screening, and inclusion of record

The breakdown of the identification, screening, and inclusion of records is noted in the PRISMA flow chart in Fig. 1.

### Evidence screening and selection process

The Rayyan intelligent systematic review software was used to collate, manage, and screen articles retrieved from the search engines. An initial screening was conducted by three independent reviewers (DA, BKK, and IAK), using article titles and abstracts, followed by a detailed review of the full-text articles selected for the review. Disagreements were resolved through discussions and consensus-building among the research team.

### Data extraction and analysis

An Excel sheet was developed to capture key information from each study included in the review, including bibliographic details (paper title, author, year), methodology, study discipline, region of study, conceptualisations of gender, findings related to malaria and gender, and policy implications or recommendations. Three researchers reviewed each article independently to ensure the consistency and reliability of data extraction. The researchers resolved discrepancies through discussions. A thematic analysis approach was employed to synthesise the findings across studies. Reviewers read each article multiple times to become deeply familiar with the content and context. Codes and themes related to gender conceptualisation were developed using the domains of gender analysis advanced by Morgan, Davies [18]. The themes were synthesised narratively, highlighting similarities, differences, and trends over time or by region, discipline, and methodological orientation.

### Data presentation

The findings from the review were guided by the gender analysis matrix advanced by Morgan, Davies [18], which highlights six gender analysis domains: sex and/or gender-disaggregated data; access to resources; distribution of labour; practices and roles; norms, values and beliefs; decision-making power; and policies, laws, and institutions. Morgan, Davies [18] explored these gender analysis domains regarding how they influence vulnerability to disease/illness exposure, response to illness/treatment, health system facilities and infrastructure, and economic, social, and security impacts of illness. The Morgan, Davies [18] Gender Analysis Framework was chosen because it offers a robust, theoretically grounded, and empirically validated approach to analysing gender dynamics within global health research, making it particularly well-suited for examining how gender has been conceptualised in malaria and gender research (see for instance, Kretchy et al. 2025). This framework adopts an intersectional, relational, and context-sensitive perspective to uncover how power, resources, roles, and norms shape gendered experiences in health systems—components that are crucial for understanding the complex interplay between gender and malaria burden, prevention, and treatment [18]. Moreover, the framework is distinguished by its flexibility and adaptability to various health contexts and research designs, including qualitative, quantitative, and mixed-methods studies. This makes it especially useful for synthesizing diverse types of malaria research while maintaining analytical coherence [18].

This review’s findings were restricted to the body of published peer-reviewed research on malaria and gender. Therefore, data from other sources that discuss works on the nexus of malaria and gender but are not published, such as grey literature, are excluded from this study. Furthermore, it’s possible that the included studies in the review did not explicitly explore gender aspects in their studies using the gender analysis matrix proposed initially by Morgan and colleagues for infectious disease outbreaks. It is, therefore, acknowledged that the results may be limited due to the analytical approach employed. That is, a different framework may yield a different outcome. However, since social, cultural, and institutional gender disparities affect the gender dimensions of malaria activities, we decided to use Morgan and colleagues’ framework for this study. It is worth noting that this gender matrix in our research was used solely as a comprehensive analytical framework to analyse how gender is applied in the studies reviewed.

The data were categorised based on country/region, fields/disciplines, research method, and the key domains for gender analysis expounded by Morgan, Davies [18]. The data were colour-coded, sorted, and summarized using tables and charts.

## Results

### Country/ region of reviewed papers

Most (66.7%) of the publications on malaria and gender focused on Africa, and only a few are on Asia (19.3%), South America (3.5%), North America (1.8%), the Caribbean (1.8%), Australia (1.8%), Europe (1.8%) and global studies (3.5%). The majority of the documents on Africa were on Nigeria (42.1%), followed by Ghana (10.5%) and Kenya (7.9%). There were two papers (5.3%) each on Malawi and Ethiopia and one paper (2.6%) each from Mozambique, Mali, Equatorial Guinea, Cote d’Ivoire, Sudan, Uganda, and Tanzania. About (7.9%) of the published works focused on Sub-Saharan Africa as a unit. For Asia, the papers were from Pakistan (45.5%), India (18.1%), Indonesia (18.1%), Myanmar (9.1%), and Yemen (9.1%). Table 1 shows the regions and countries covered in the reviewed studies.

**Table 1:** Region/Country of reviewed papers

Country/Region	Frequency	Percentage
Africa (Nigeria, Ghana, Malawi, Ethiopia, Kenya, Sudan, Uganda, Tanzania, Mozambique, Burkina Faso, Mali, Cote d’Ivoire, Equatorial Guinea)	38	66.7
Asia (Yemen, India, Myanmar, Pakistan, Indonesia)	11	19.3
North America (US)	1	1.8
South America (Brazil)	2	3.5
Caribbean (Jamaica)	1	1.8
Australia	1	1.8
Europe (UK)	1	1.8
Global/General	2	3.5
**Grand Total**	**57**	**100**

### Discipline of study

Out of the 57 studies reviewed, 34 (59.6%) were biomedical science papers, 4 (7.0%) were social science papers, and 19 (33.3%) were multi-disciplinary research. Out of the biomedical papers, 22 (64.7%) focused on microbiology and genetics, mostly involving laboratory research, while 12 (35.3%) were from the field of public health, mainly epidemiology. The multi-disciplinary studies combined social science (sociology, gender studies, and economics) with public health research (environmental science, vector control, and surveillance). Table 2 shows the breakdown of the reviewed studies by year of publication, country, discipline, and research approach.

### Research approach/study type

**Fig. 2:**
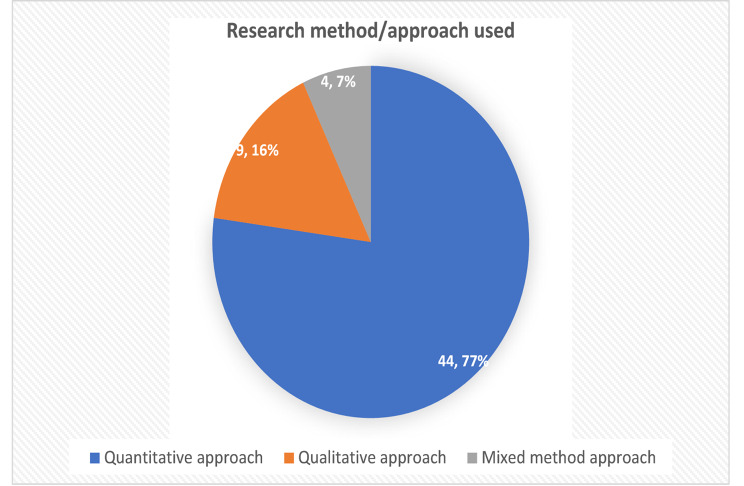
Research method/approach used

Most (77.2%, 44) of the studies reviewed employed a quantitative research approach. These studies were either hospital- or community-based and relied on primary or secondary data. Approximately 16% [9] of the reviewed studies employed a qualitative research methodology, relying mainly on primary data for their analysis. A few of the studies (7%, 4), however, employed a mixed-methods research approach, utilising primary data collected at the community level. Figure 2 shows the research method/approach used in the reviewed studies.

**Table 2:** Breakdown of the reviewed studies by year of publication, country, discipline, and research approach

Article Title and Author(s)	Year of Publication	Country/ Region	Discipline Of Study	Research Method/Study Type
Edstein, Nasveld [24]	2007	Australia	Biomedical Science / Laboratory Research	Quantitative / Hospital-Based Study
Maraka, Akala [25]	2020	Kenya	Biomedical Science / Laboratory Research	Quantitative / Hospital-Based Study
Vieira, Mello [26]	2020	Brazil	Biomedical Science / Laboratory Research	Quantitative / Hospital-Based Study
Dhangadamajhi, Kar [27]	2009	India	Biomedical Science / Laboratory Research	Quantitative / Hospital-Based Study
Morris, Tan [28]	2013	US	Biomedical Science / Laboratory Research	Quantitative / Clinical Trial
Maiga, Opondo [29]	2022	Mali	Biomedical Science / Laboratory Research	Quantitative / Randomized Controlled Trial
Segata, Baldini [30]	2016	Burkina Faso	Biomedical Science / Laboratory Research	Laboratory Experiment
Ogbonna, Ezeoru [31]	2021	Nigeria	Biomedical Science / Laboratory Research	Quantitative (A Cross-Sectional Study)
Ezenwa [32]	2023	Nigeria	Biomedical Science / Laboratory Research	Quantitative (Community-Based Study)
Jeremiah, Eze [33]	2021	Nigeria	Biomedical Science / Laboratory Research	Quantitative/Randomized Controlled Trial
Rafique, Hussain [34]	2022	Pakistan	Biomedical Science / Laboratory Research	Quantitative (Cross-Sectional Study)
Sansan [35]	2016	Cote d’Ivoire	Biomedical Science / Laboratory Research	Quantitative (Hospital- Based Study)
Zeb, Irshad [36]	2022	Pakistan	Biomedical Science / Laboratory Research	Quantitative (Hospital- Based Study)
Raasti, Nasir [37]	2024	Pakistan	Biomedical Science / Laboratory Research	Descriptive Cross-Sectional Study
Neboh and Okaka [38]	2019	Nigeria	Biomedical Science / Laboratory Research	Quantitative
Onwuzurike, Nkpeh [39]	2023	Nigeria	Biomedical Science / Laboratory Research	Quantitative (Hospital- Based Study)
Esan, Omisakin [40]	2014	Nigeria	Biomedical Science / Laboratory Research	Quantitative (Hospital- Based Study)
ur Rehman, Khan [41]	2022	Pakistan	Biomedical Science / Laboratory Research	Quantitative (Observational / Cross-Sectional Study)
Ojo, Jonathan [42]	2022	Nigeria	Biomedical Science / Laboratory Research	Quantitative/Retrospective Study
Esan, Omisakin [43]	2014	Nigeria	Biomedical Science / Laboratory Research	Quantitative (Hospital- Based Study)
Ayodele [44]	2014	Nigeria	Biomedical Science / Laboratory Research	Quantitative (Hospital- Based Study)
Wedekind, Walker [45]	2006	UK	Biomedical Science / Laboratory Research	Laboratory Experiment
Quaresima, Agbenyega [46]	2019	Ghana	Epidemiology	Mixed Methods (Primary Data)
DeBoer, Vaz [47]	2023	Equatorial Guinea	Social Science / Public Health (Vector Control & Surveillance, Gender Studies)	Quantitative / Secondary Data
Olapeju, Choiriyyah [48]	2018	SubSaharan Africa	Epidemiology	Quantitative / Secondary Data
Rose, Ashfaq [49]	2018	Pakistan	Social Science (Agric-Economics)	Quantitative / Secondary Data
Quaresima, Agbenyega [9]	2021	Ghana	Social Science / Public Health (Sociology, Gender Studies)	Mixed Method/Hospital and Community-Based Data
Emmanuel Okoro, Ifeanyichukwu Romanus [50]	2023	Nigeria	Epidemiology	Quantitative / Hospital-Based Study
Willis and Hamon [51]	2018	Africa	Social Science / Public Health (Agric- Economics, Sociology, Gender Studies)	Quantitative / Secondary Data
Mohamedani, Mirgani [52]	1996	Sudan	Social Science / Public Health (Gender Studies)	Quantitative / Community-Based Study
Wai [53]	2001	Myanmar	Social Science / Public Health (Sociology, Gender Studies)	Qualitative / Community-Based Study
Rawlings [54]	2016	Brazil	Social Science / Public Health (Sociology, Health Economics)	Quantitative / Secondary Data
Okiring, Epstein [55]	2022	Uganda	Epidemiology	Quantitative / Hospital-Based Study
Onyango and Maguire [56]	2022	Kenya	Social Science / Public Health (Environmental / Sociology, Gender Studies)	Qualitative/Community- Based Study
Austin, Noble [57]	2014	90 less-developed countries	Social Science / Public Health (Health Economics, Sociology, Gender Studies)	Quantitative / Secondary Data
Tolhurst, Amekudzi [58]	2008	Ghana	Social Science / Public Health (Sociology, Gender Studies)	Qualitative / Community- Based Study
Diiro, Kassie [59]	2022	Ethiopia	Social Science (Agric-Economics)	Quantitative / Community Level Study / Primary Data
Alubabari and Aborlo [60]	2011	Nigeria	Social Science / Public Health (Sociology, Gender Studies)	Qualitative / Literature Review
Woldu and Haile [10]	2015	Kenya	Social Science (Anthropology)	Mixed Method / Community-Based Data
Klein, Barham [61]	2019	Malawi	Social Science (Agric-Economics)	Quantitative / Secondary Data
Minja, Tanner [62]	2001	Tanzania	Social Science / Public Health (Anthropology, Gender Studies)	Quantitative / Community Level Study / Primary Data
Tanner and Vlassoff [63]	1998	General/Global	Social Science / Public Health (Sociology / Gender Studies)	Qualitative / Conceptual
Hildon, Escorcio-Ymayo [64]	2022	Mozambique	Social Science / Public Health (Sociology, Gender Studies)	Qualitative / Community-Based Study
Diiro, Affognon [8]	2016	Kenya	Social Science / Public Health (Sociology / Gender Studies)	Quantitative / Community-Based Study
Sumriati, Tosepu [65]	2022	Indonesia	Epidemiology	Quantitative / Secondary Data
Willis and Hamon [66]	2005	Sub-Saharan Africa	Social Science / Public Health (Agric-Economics, Sociology/Gender Studies)	Qualitative / Literature Review
Tolhurst and Nyonator [67]	2005	Ghana	Social Science / Public Health (Sociology, Gender Studies)	Qualitative / Hospital-Based Study
Kunihya, Samaila [68]	2016	Nigeria	Epidemiology	Quantitative / Hospital- Based Study
Garley, Ivanovich [69]	2013	Nigeria	Social Science / Public Health (Epidemiology, Gender Studies)	Quantitative / Community Level Study / Primary Data
Xing, Zhang [70]	2024	Nigeria	Epidemiology	Quantitative/Secondary Data
Gray [71]	2013	Jamaica	Social Science / Public Health (Sociology, Gender Studies)	Advocacy Piece / Quantitative
Nas, Yahaya [72]	2017	Nigeria	Epidemiology	Quantitative / Hospital-Based Study
Al-Taiar, Chandler [73]	2009	Yemen	Social Science / Public Health (Sociology/Gender Studies)	Mixed-Method / Community-Based Study
Ayele, Zewotir [74]	2012	Ethiopia	Epidemiology	Quantitative / Secondary Data
Simwaka, Makwiza [75]	2006	Malawi	Epidemiology	Qualitative / Community- Based Study
Tripathy, Mohanty [76]	2016	India	Epidemiology	Quantitative / Community-Based Study
Oktafandi and Sungkar [77]	2015	Indonesia	Epidemiology	Quantitative / Hospital-Based Study

### Year of publication of reviewed studies

Although the article search focused on all existing published research on malaria and gender, the articles retrieved and reviewed were those published between 1995 and 2024. The majority of the papers were published after 2012, and no papers were published in 1995. Table 3 shows the year of publication of the reviewed studies.

**Table 3:** Year of publication of reviewed studies

Year of publication	Frequency	Percentage	Year of publication	Frequency	Percentage
1996	1	1.8	2014	4	7.0
1998	1	1.8	2015	2	3.5
2001	2	3.5	2016	6	10.5
2005	2	3.5	2017	2	3.5
2006	2	3.5	2018	2	3.5
2007	1	1.8	2019	3	5.3
2008	1	1.8	2020	2	3.5
2009	2	3.5	2021	3	5.3
2011	1	1.8	2022	10	17.5
2012	1	1.8	2023	4	7.0
2013	3	5.3	2024	2	3.5

### Application of gender analysis domains in the reviewed studies

Regarding the number of gender analysis domains applied, only 7.0% of the reviewed papers have all the six [6] gender analysis domains proposed by Morgan, Davies [18]. About 16% of the papers applied five [5] of the domains, 8.8% applied four [4] of the domains, and another 8.8% of the papers applied only three [3] domains. About 11% of the reviewed papers applied two [2] of the domains, while 47.3% of the papers applied only one [1] domain. All the reviewed studies applied the sex-/gender disaggregated data domain; 49.1% applied the domain covering the distribution of labour, practices, and roles, while 40.4% of the studies applied the domain covering norms, values, and beliefs. However, only 31.6% discussed access to resources, 26.3% applied the decision-making power and autonomy domain, and 12.3% applied the domain covering policies, laws, and institutions. Table 4 shows the gender analysis domains applied in the reviewed study.

**Table 4:** Gender analysis domains applied in the reviewed study

Reviewed Papers	Gender analysis domains						
	Sex-/gender disaggregated data	Access to resources	Distribution of labour, practices, roles	Norms, values, beliefs	Decision-making power, autonomy	Policies, laws, institutions	Number of domains applied in each paper
Onwuzurike, Nkpeh [39]	x						1
DeBoer, Vaz [47]	x	x	x	x	x	x	6
Edstein, Nasveld [24]	x						1
Quaresima, Agbenyega [9]	x		x				2
Esan, Omisakin [43]	x						1
Mohamedani, Mirgani [52]	x	x	x	x	x	x	6
Maraka, Akala [25]	x						1
Wai [53]	x		x	x			3
Neboh and Okaka [38]	x						1
Oktafandi and Sungkar [77]	x		x				2
Sumriati, Tosepu [65]	x	x	x	x			4
Rawlings [54]	x	x					2
Ayele, Zewotir [74]	x	x	x	x			4
Okiring, Epstein [55]	x		x				2
Kunihya, Samaila [68]	x						1
Simwaka, Makwiza [75]	x	x	x	x	x		5
Garley, Ivanovich [69]	x						1
[76]	x		x	x			3
Onyango and Maguire [56]	x		x	x	x		4
Vieira, Mello [26]	x						1
Austin, Noble [57]	x	x	x	x	x	x	6
Dhangadamajhi, Kar [27]	x						1
Tolhurst, Amekudzi [58]	x	x	x	x	x	x	6
Morris, Tan [28]	x						1
Quaresima, Agbenyega [46]	x		x				2
Emmanuel Okoro, Ifeanyichukwu Romanus [50]	x						1
Rose, Ashfaq [49]	x		x	x			3
Diiro, Kassie [59]	x		x	x		x	4
Minja, Tanner [62]	x						1
Alubabari and Aborlo [60]	x	x	x	x		x	5
Maiga, Opondo [29]	x						1
Woldu and Haile [10]	x	x	x	x			4
Klein, Barham [61]	x				x	x	3
[62]	x	x	x	x	x		5
Segata, Baldini [30]	x						1
Tanner and Vlassoff [63]	x	x	x	x	x		5
Ogbonna, Ezeoru [31]	x						1
Olapeju, Choiriyyah [48]	x		x				2
Nas, Yahaya [72]	x						1
Hildon, Escorcio-Ymayo [64]	x	x	x	x	x		5
Ezenwa [32]	x						1
Wedekind, Walker [45]	x						1
Diiro, Affognon [8]	x	x	x	x	x		5
Ayodele [44]	x						1
Jeremiah, Eze [33]	x						1
Willis and Hamon [66]	x	x	x	x	x		5
Esan, Omisakin [40]	x						1
Rafique, Hussain [34]	x						1
Tolhurst and Nyonator [67]	x	x	x	x	x		5
Sansan [35]	x						1
Xing, Zhang [70]	x	x	x	x	x		5
Zeb, Irshad [36]	x						1
Ojo, Jonathan [42]	x						1
Gray [71]	x		x	x			3
Raasti, Nasir [37]	x						1
ur Rehman, Khan [41]	x						1
Al-Taiar, Chandler [73]	x	x	x	x	x		5
**Total number of papers applying the various domains**	57	18	28	23	15	7	

x indicates the presence of a specified gender analysis domain for the article

## Discussion

The review reveals a paucity of published research studies on malaria and gender. Most existing research has focused on Africa, which is not surprising, given that the African region bears the highest malaria burden, with Nigeria recording the highest number of cases [1]. The majority of the published papers on malaria and gender have been biomedical, reducing gender analysis to only sex-disaggregated data and using sex and gender interchangeably. There is a notable lack of social science research on malaria and gender. Meanwhile, social science research offers an avenue for conducting a thorough gender analysis using all six gender analysis domains advanced by Morgan, Davies [18]. Moreover, most existing published work on malaria and gender employed a quantitative research approach, with the majority of the studies being laboratory-based. There were very few community-based studies that employed qualitative research methodologies and data collection strategies such as focus group discussions (FGDs) and in-depth interviews. There were also very few studies that used a mixed-methods research approach. The dominance of biomedical and epidemiological approaches, often at the expense of qualitative and participatory methodologies, limits the depth of gender analysis. Integrating feminist, anthropological, and community-based research methods could enrich the understanding of how gender norms and power relations shape malaria experiences and outcomes.

Regarding the application of gender analysis domains, it was found that all the reviewed studies have applied the basic domain of gender analysis, which is sex-disaggregated data. The sex-disaggregated data showed the gender differences in malaria incidence, knowledge, prevention, treatment, and impact. For instance, malaria incidence was higher among women of childbearing age compared to their male counterparts [55, 68, 72]. Based on household circumstances, more females and children used insecticide-treated nets than males [48, 69]. Additionally, males demonstrated significantly better knowledge about malaria despite being less involved in caring for the sick members of their household [8, 50, 76]. Malaria has a greater impact on women’s agricultural productivity than on men’s, mainly due to caregiving roles [10, 59, 66].

Gender norms, values, and beliefs were primarily discussed in conjunction with the distribution of labour, practices, and roles in the reviewed studies [9, 10, 53, 56]. These studies highlight how gender roles and gender division of labour based on gender norms and beliefs expose men and women differently to malaria infections and malaria prevention activities. For instance, women who mostly trade in the night had higher malaria infection rates than men while household chores that are performed in the evening or night expose women to malaria bites [9, 56, 73]. Regarding malaria prevention activities, women are responsible for carrying out tasks to prevent malaria, such as burning mosquito coils at night and washing and hanging bed nets [53]. Culturally prescribed gender roles in agricultural communities play important roles in explaining the disparity in reported malaria incidence among men and women [10]. Gender roles also determine who is responsible for providing care for sick relatives. At the same time, gender norms and beliefs place decision-making power and authority as well as the control of family resources in the hands of men, leaving women to rely on men for decisions and resources to seek treatment for sick members of the household [70, 73].

Access to resources, decision-making power and autonomy were discussed together in relation to prevailing gender norms [56, 59]. Gender norms generally place family resources in men’s care, which also influences the prerogative to make decisions regarding where and when to seek treatment for sick members of the household [56, 59]. However, where women have access to personal resources, they can decide when and where to seek treatment, especially for their sick children. Women’s access to resources also impacts how they prevent and cope with malaria [57]. Women’s increased economic autonomy has been linked to their access to social and health resources that reduce malaria transmission, such as acquiring preventive devices like mosquito nets and repellent [57]. Increased empowerment in terms of decision-making autonomy also decreases the likelihood of contracting malaria within the household [61]. Malaria also has direct and indirect effects on agricultural productivity among smallholder farmers, especially female farmers, due to time spent caring for sick relatives, which further reduces their access to resources [56, 59].

Discussions around policies, institutions, and laws centred on equipping women to function effectively in malaria prevention, treatment, and control [47, 52, 58, 61]. For instance, the importance of women’s role in vector control has been emphasized. There is advocacy for the participation of women in indoor residual spraying (IRS) campaigns, as well as a renewed effort to implement equitable policies and practices that intentionally engage women in vector control activities [47].

This systematic descriptive review reveals significant gaps and trends in the conceptualization and application of gender in malaria research, offering critical implications for theory and practice. Theoretically, the findings demonstrate the persistent conflation of “sex” and “gender” in much of the literature, underscoring the need for a more nuanced and intersectional approach to gender analysis. Practically, the underutilization of comprehensive gender analysis impedes the development of equitable and effective malaria interventions. For instance, overlooking gendered patterns in labour, mobility, and healthcare access may lead to interventions that fail to reach vulnerable populations like women, adolescent girls, and other marginalized groups.

## Conclusion

This systematic descriptive review provides a timely and in-depth analysis of how gender is addressed in malaria research, examining its conceptualization, operationalization, and methodological use across various disciplines and regions. Despite increased awareness of gender’s impact on health outcomes, few studies have critically explored how it is framed within malaria-related literature. Meanwhile, the majority of the published papers on malaria and gender have been biomedical in nature, reducing gender analysis to only sex-disaggregated data. Moreover, most studies employed a quantitative research approach, with the majority being laboratory-based. The existing published research on malaria and gender has paid little attention to women in malaria control such as community health workers and the impact of malaria on adolescent girls.

More social science research that employs qualitative, mixed-methods and community-based approaches to gender and malaria research is needed. Increased effort is recommended towards academic research and evidence production on malaria and gender, employing detailed gender analysis. These approaches extend gender analysis beyond sex and/or gender-disaggregated data, and includes other domains, such as access to resources; distribution of labour; practices and roles; norms, values and beliefs; and decision-making power. Increased evidence on the gender dimensions of malaria, coupled with the right policies and advocacy, will lead to gains in controlling and eventual eradication of malaria and achieving gender equality.

## Data Availability

All the relevant data used in the analysis have been included in this paper by providing the search strategy used, the list of the included studies, the data extraction procedure and analysis, and the criteria for the selection of studies.

## Abbreviations

FGD: Focus Group Discussion
LMIC: Low- and Middle-Income Countries
PRISMA: Preferred Reporting Items for Systematic Reviews and Meta-Analyses
SSA: sub-Saharan Africa
WHO: World Health Organisation
WMR: World Malaria Report
